# Successful Treatment of a Case of Metallo-Beta-Lactamase-Producing *Raoultella ornithinolytica* Bacteremia by Antimicrobial Stewardship Team Intervention and Therapeutic Drug Monitoring-Based Amikacin Treatment

**DOI:** 10.1155/2023/5574769

**Published:** 2023-04-07

**Authors:** Noriko Koishi, Hiroshi Sasano, Toshihiro Yoshizawa, Mika Shikuri, Hiroshi Matsumoto, Mai Suzuki, Yukiko Fukui, Masayoshi Chonan, Toshimi Kimura, Hirofumi Ichida, Akio Saiura, Toshio Naito

**Affiliations:** ^1^Department of Pharmacy, Juntendo University Hospital, Tokyo, Japan; ^2^Department of General Medicine, Juntendo University Faculty of Medicine, Tokyo, Japan; ^3^Department of Clinical Laboratory, Juntendo University Hospital, Tokyo, Japan; ^4^Department of Hepatobiliary-Pancreatic Surgery, Juntendo University Faculty of Medicine, Tokyo, Japan

## Abstract

An 80-year-old woman underwent pancreatoduodenectomy. Post-operation, she experienced a fever, and a culture of blood revealed metallo-beta-lactamase-producing *Raoultella ornithinolytica.* For treatments with aminoglycoside antimicrobial agents, a therapeutic drug monitoring-based dosing design can lower the risk of adverse events and enable appropriate treatment. *Key Clinical Message*. When aminoglycoside antimicrobial agents are administered for MBL-producing bacteremia, prescription suggestions based on TDM by antimicrobial stewardship team can reduce the occurrence of adverse events and enable appropriate treatment.

## 1. Introduction

The genus *Raoultella* consists of Gram-negative, oxidase-negative, catalase-positive, aerobic, and nonmotile rods that belong to the Enterobacteriaceae family [1]. Originally, it was classified in the genus *Klebsiella*, but based on comparative analysis of 16S rRNA and rpoB genes, a new genus *Raoultella* was established in the current classification system. Although bloodstream infections caused by *Raoultella ornithinolytica* within the genus *Raoultella* are rare, there have been several reports of *R. ornithinolytica* bacteremia [2–4].

In the antimicrobial stewardship team (AST) at Juntendo University Hospital, blood culture rounds are conducted, and infectious disease specialists and pharmacists collaborate to recommend antimicrobial agents based on drug sensitivity test results and design prescriptions based on therapeutic drug monitoring (TDM) [5, 6].

Herein, we report a case in which the AST intervened and successfully treated multidrug-resistant *R. ornithinolytica* bacteremia that was resistant to piperacillin/tazobactam, fluoroquinolone, and carbapenem by administering amikacin (AMK) based on TDM.

## 2. Case History/Examination

The patient was an 80-year-old female. In June 20XX-1, she was diagnosed with an intraductal papillary mucinous tumor of the main pancreatic duct type due to dilatation of the main pancreatic duct and was kept under observation. In May 20XX, she was diagnosed with adenocarcinoma by endoscopic retrograde cholangiopancreatography. Subsequently, she was admitted to the hospital in June 20XX for perioperative glycemic control and a pancreaticoduodenectomy for the intraductal papillary mucinous tumor. Her past medical history included colorectal cancer (post endoscopic surgery), right breast cancer, diabetes mellitus, hypertension, and hyperlipidemia, and she had an allergy to iodine-based contrast media.

## 3. Differential Diagnosis, Investigations, and Treatment

On physical examination, her consciousness was clear, temperature was 36.1°C, pulse was 73 bpm, respiratory rate was 14 breaths-/min, blood pressure was 123/53 mmHg, and SpO_2_ was 98% in room air. The patient's respiration was clear, heart sounds were regular and there was no murmur, the abdomen was flat and soft, and she showed normal bowel peristalsis.

On the 8th day of hospitalization (July 6), pancreatic cancer resection was performed. On the second postoperative day (July 8), a fever of 38.2°C and shivering were observed. Suspecting cholangitis, a blood culture was performed, and administration of meropenem (MEPM) at a dose of 0.5 g every 8 h was started. On the third postoperative day (July 9), the dose of MEPM was increased to 1 g every 8 h, and Gram-negative rods were detected in two of two sets of blood cultures. On the fourth postoperative day (July 10), the bacterium was identified to be *R. ornithinolytica*, and the same species was also identified from bile cultures. At this point, since the bacterium showed resistance to imipenem on antibiotic susceptibility testing by the disk method, carbapenems were considered to be ineffective, and the AST recommended discontinuing MEPM; however, as the bacterium showed susceptibility to AMK, the AST recommended starting AMK at 560 mg (12 mg/kg) once every 24 h. Subsequently, the bacterium was also found to be resistant to piperacillin/tazobactam, and it was finally identified to be metallo-beta-lactamase (MBL)-producing *R. ornithinolytica* by sodium mercaptoacetic acid disk method and modified carbapenem inactivation method (mCIM). Moreover, drug-resistance genes were amplified by PCR assay and identified as NDM type. Four days after the start of AMK administration (postoperative day 7; July 13), the concentration of AMK in blood showed a peak at 49.1 *μ*g/mL and a trough at 2.1 *μ*g/mL. On postoperative day 9 (July 15), a fever of 37.6°C and an elevated C-reactive protein level were observed, and the administration of 1 g of MEPM every 8 h was resumed after blood, urine, and sputum were cultured.

## 4. Outcome and Follow-Up

**Figure 1:**
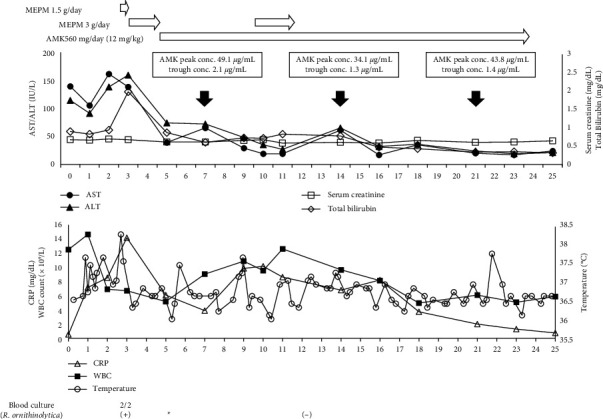
The patient's clinical course. MEPM: meropenem; AMK: amikacin. The clinical course is shown with the day of surgery as day 0. ^*∗*^Identified to be a MBL producer.

On postoperative day 10 (July 16), blood, urine, and sputum cultures revealed no significant bacteria, and MEPM was discontinued on postoperative day 11 (July 17). AMK administration was continued throughout this period, and the concentration of AMK in blood on the 18th day of AMK administration (postoperative day 21; July 27) showed a peak at 43.8 *μ*g/mL and a trough at 1.4 *μ*g/mL. AMK administration was terminated on postoperative day 23 (July 29) after a total of 20 days (12 days after the negative blood culture). Hearing tests were performed during the AMK administration period, and no abnormalities were found; in addition, there was no decline in renal function during the AMK administration period (Figure 1).

**Figure 2:**
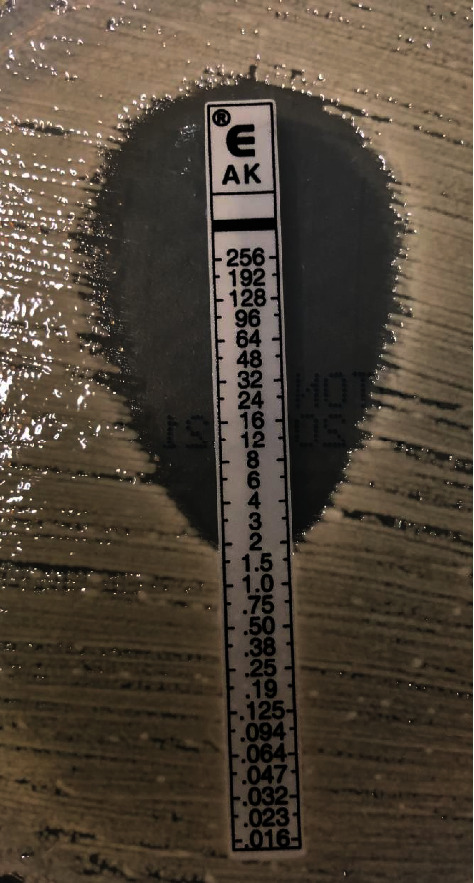
Results of AMK drug susceptibility test of *R. ornithinolytica* by *e*-test.

The minimum inhibitory concentration (MIC) of AMK in the detected bacteria was ≤8 *μ*g/mL by the liquid microdilution method. In a subsequent *E*-test, the MIC was 2 *μ*g/mL (Figure 2). After that, the general condition was confirmed to be stable without recurrence of bacteremia, and she was discharged on postoperative day 41 (August 16).

## 5. Discussion

In recent years, infections caused by multidrug-resistant microorganisms have become a frequent problem in Japan.

Multidrug-resistant *Pseudomonas aeruginosa* and multidrug-resistant *A. baumannii* produce MBLs that degrade a wide range of beta-lactams, including carbapenems, making the treatment of infections caused by these organisms difficult [7]. In cases of infection involving multidrug-resistant strains, aminoglycosides, such as AMK, are among the few antimicrobial agents that remain useful against the majority of Gram-negative bacteria.

*Raoultella* strains are usually susceptible to beta-lactamase-containing penicillin, amoxicillin, and third- and fourth-generation cephalosporins, carbapenems, fluoroquinolones, and aminoglycosides [8], and previously reported cases of bacteremia were treated with these antimicrobial agents. However, these cases were not MBL-producing bacterium, and also, there was no description of blood concentrations in cases treated with aminoglycoside [2–4]. Cases of MBL-producing bacterium detected at surgical sites were treated with tigecycline rather than aminoglycosides [9].

In this case, the detected *R. ornithinolytica* was an MBL-producing bacterium resistant to piperacillin/tazobactam and carbapenems; whereas, it was susceptible to AMK and fluoroquinolones. However, fluoroquinolones were not an option in this case because ciprofloxacin-resistant *Enterobacter cloacae* were detected in a bile culture taken on a different day than the blood culture. According to the severity classification of medical guidelines [10], the severity of the cholangitis in this case was moderate. In the Japan TDM guideline [11], the target peak concentration of AMK in moderate Gram-negative bacillus infections is from 41 to 49 *μ*g/mL, and the trough concentration is less than 4 *μ*g/mL. All of the measured blood concentrations of AMK over the treatment course were within the reference values, and we were thus able to continue the treatment without any change in the dosage.

Characteristic side effects of AMK are renal impairment and hearing impairment. It is known that the incidence of renal impairment is correlated with the trough levels of AMK in blood [12]. It has also been reported that renal dysfunction is not affected by AMK treatment for over 10 days, with once daily administration [13]. In this case, no renal dysfunction was observed despite the administration period of more than 10 days; this may have been due to the control of the trough concentration in blood by the TDM. On the other hand, hearing impairment is considered to be an irreversible adverse event, and its occurrence is correlated with the total dose; thus, it must be considered during long-term AMK administration [14]. In this case, an otolaryngology consultation was recommended and a hearing test was performed, but no abnormalities were found. We believe that a hearing test should be performed at least once during AMK treatment to avoid the inability to choose to AMK due to the onset of hearing impairment.

The appropriate duration of treatment for bloodstream infections caused by Gram-negative rods has not been clearly defined [15]. The duration of treatment is determined by clinical judgment based on various factors, such as the clinical response, identification of the causative organism, control of the source of infection, and the patient's immunological status [16]. It has been reported that follow-up blood culture for bacteremia caused by Gram-negative rods leads to the wastage of resources, *e.g*., false positive blood cultures, additional tests, prolonged duration of antimicrobial administration, and prolonged hospital stay [17]. On the other hand, it has been reported that follow-up blood culture should be performed for multidrug-resistant bacteria [18]. In this case, we recommended follow-up blood culture based on the fact that the causative bacterium was multidrug-resistant. As for the clinical effects, we observed a reduction in the fever and a decrease in the inflammatory response and confirmed a negative blood culture. We were able to terminate the AMK treatment after a total of 20 days of treatment; thereafter, the patient was discharged without any relapse of bacteremia. Although aminoglycoside antimicrobials are used in a conservative manner due to their adverse events, Bartal et al. reported that TDM resulted in less nephrotoxicity and improved clinical outcomes [19]. Since TDM can be performed safely even in late-stage elderly patients, such as this case, to guide the appropriate selection of prescriptions and tests, AST intervention plays an important role in improving patient outcomes.

In cases such as in situation with fewer resources where aminoglycosides are administered without TDM, we recommend creating protocols for prescribing, administrating, and monitoring (e.g. renal function and hearing test) for optimal dosage to minimize adverse events.

In addition, the liquid microdilution method could only determine that the MIC of AMK was ≤8 *μ*g/mL. This is because the minimum concentration of amikacin drug susceptibility panel used in the liquid microdilution method at our hospital laboratory is 8 *μ*g/mL, and the concentration range below this cannot be measured. The *E*-test performed afterwards determined that the MIC was ≤2 *μ*g/mL. For aminoglycosides, relevant pharmacokinetic/pharmacodynamic (PK/PD) parameters that can predict bacterial killing and the clinical response are the peak serum concentration (Cpeak) and the MIC of the agent in the target pathogen, more specifically, a ratio of the Cpeak to the MIC (Cpeak/MIC) of ≥8 [20, 21].

In this case, the Cpeak/MIC calculated based on the liquid microdilution method did not reach the target value of 8. In contrast, the Cpeak/MIC calculated based on the *E*-test exceeded the target value, so it was considered that the effect of AMK was maximal, and the dose did not need to be increased. Relying only on the results based on the liquid microdilution method risks unnecessarily increases the dose and causes adverse events in the patient. By using the *E* test, it is possible to measure the MIC more easily and precisely. *E*-tests can be used to measure MICs that are difficult to measure with routine tests and to guide appropriate treatment selection based on the PK/PD parameters of individual patients.

In conclusion, when aminoglycoside antimicrobial agents are administered for MBL-producing *R. ornithinolytica* bacteremia associated with biliary tract infection, AST intervention and prescription suggestions based on TDM can reduce the occurrence of adverse events and guide the selection of appropriate treatment.

## Data Availability

No data were used to support the findings of this study.
